# Endophytic fungi from the Amazonian plant *Paullinia cupana* and from *Olea europaea* isolated using cassava as an alternative starch media source

**DOI:** 10.1186/2193-1801-2-579

**Published:** 2013-10-30

**Authors:** Eliandra de Freitas Sia, Joelma Marcon, Danice Mazzer Luvizotto, Maria Carolina Quecine, Sarina Tsui, José Odair Pereira, Aline Aparecida Pizzirani-Kleiner, João Lúcio Azevedo

**Affiliations:** Universidade Federal do Amazonas-UFAM, Avenida Rodrigo Otávio Ramos 3000,Colorado I, Manaus, 69.077-000 AM Brazil; Escola Superior de Agricultura “Luiz de Queiroz”, Department of Genetics, University of São Paulo, Av. Pádua Dias 11, PO BOX 83, 13400-970 Piracicaba, SP Brazil

**Keywords:** Olive, Guarana, Potato, Cassava, *Manihot esculenta*

## Abstract

Endophytic fungi live inside plants, apparently do not cause any harm to their hosts and may play important roles in defense and growth promotion. Fungal growth is a routine practice at microbiological laboratories, and the Potato Dextrose Agar (PDA) is the most frequently used medium because it is a rich source of starch. However, the production of potatoes in some regions of the world can be costly. Aiming the development of a new medium source to tropical countries, in the present study, we used leaves from the guarana (a tropical plant from the Amazon region) and the olive (which grows in subtropical and temperate regions) to isolate endophytic fungi using PDA and Manihot Dextrose Agar (MDA). Cassava (*Manihot esculenta*) was evaluated as a substitute starch source. For guarana, the endophytic incidence (EI) was 90% and 98% on PDA and MDA media, respectively, and 65% and 70% for olive, respectively. The fungal isolates were sequenced using the ITS- rDNA region. The fungal identification demonstrated that the isolates varied according to the host plant and media source. In the guarana plant, 13 fungal genera were found using MDA and six were found using PDA. In the olive plant, six genera were obtained using PDA and 4 were obtained using MDA. The multivariate analysis results demonstrated the highest fungal diversity from guarana when using MDA medium. Interestingly, some genera were isolated from one specific host or in one specific media, suggesting the importance of these two factors in fungal isolation specificity. Thus, this study indicated that cassava is a feasible starch source that could serve as a potential alternative medium to potato medium.

## Background

Endophytic fungi are known to live inside plants during parts of their life cycle and apparently do not cause any harm to their hosts. They have been found in all plant hosts so far examined and may play important roles in helping their hosts producing plant growth hormones, enzymes, and siderophores, and may act against plant diseases and insect pests (Azevedo et al. 2000; Azevedo and Araújo 2007; Vega et al. 2008; Suryanarayanan et al. 2012). The study of endophytic fungi is relevant because these fungi may also be used for the production of secondary metabolites and could serve as vectors for the introduction of genes that can confer pest resistance and that could express other important traits (Stierle et al. 1993; Strobel 2003; Lacava et al. 2010).

Most endophytic fungi were first studied in plants from temperate climates. These fungi have also recently been isolated from many tropical hosts. Most study results indicate that endophytes from tropical hosts constitute a species-rich ecological assemblage of fungi (Arnold et al. 2000; Arnold and Lutzoni 2007; Rungjindamai et al. 2008; Tejesvi et al. 2009; Huang et al. 2009; Rocha et al. 2011), though this is not a general trend (Suryanarayanan et al. 2011).

A better understanding of fungal diversity may prove crucial in fungi utility and cultivation. However, fungi isolation in pure cultures has been limited. Of the estimated 1.5 million species that are thought to exist, less than 5% have been successfully isolated into pure cultures (Hawksworth 2001; O’Brien et al. 2005). Limitations in cultivation methods are thought to include the following: fast-growing fungal species outcompeting slower growing species; many fungi are not adapted to grow and sporulate in artificial culture media (Taylor et al. 2000; O’Brien et al. 2005). The development of a high-throughput, dilution-to-extinction approach has been developed for fungi that significantly increased the assessment of the diversity of cultured fungi (Collado et al. 2007). Novel approaches to culturing fungi that utilize low levels of nutrients or simulated natural environments have not yet been adopted, and the development of new media sources represents an important way for future research (Ferrari et al. 2011).

Thus, in the present study, we compared the abundance and diversity of endophytic fungi that were isolated from two tree species. The first species, *Paullinia cupana*, is a tropical plant that originated from the Brazilian Amazon forest. Its “guarana” fruits are primarily used to produce soft drinks, and it has different medicinal properties (Freitas et al. 2007). The second host used in this study was the olive plant, *Olea europaea*, which originated in temperate and subtropical regions and was introduced to the south and southwest of Brazil centuries ago; its fruits are used *in natura* and to produce olive oil (Muzzalupo et al. 2009). The primary focus of our study was that the endophytic fungi were isolated using two distinct fungal culture media: Potato Dextrose Agar (PDA), which is largely used in mycological laboratories and is derived from potato starch (*Solanum tuberosum*) and a recently introduced medium called Manihot Dextrose Agar (MDA) with Manihot, also called cassava or yuca (*Manihiot esculenta*) as an alternative source of starch (Sia 2012). *M. esculenta* is a tropical plant with high commercial importance to Brazil, Africa and other parts of the world. It is widely cultivated and could potentially be used to substitute PDA medium in tropical countries where cassava is cheaply cultivated. Our data strong suggest, by the increased number of isolates and the diversity of endophytic fungal communities using MDA that this medium is a great alternative to researchers access the immense unknown plant-associated fungal diversity.

## Results

### Fungal isolation and diversity

Both PDA and MDA media were used to isolate fungi from both the guarana and olive plants. For guarana, the EI were 90% and 98% using PDA and MDA, respectively. For the olive plant, the EI were 65% and 70% using PDA and MDA, respectively.

Sixty-one fungal isolates were obtained from guarana: 28 on PDA and 33 on MDA. Fifty-three isolates were obtained from olive: 26 on PDA and 27 on MDA. These isolates were sequenced using the ITS-rDNA region. All of the isolates were identified at the genera level and a larger amount of fungal genera was obtained from guarana than from olive (Table 1). Some genera in the endophytic fungal communities were isolated from a single host or on only a single medium. This shows the effect that these two factors have on fungal isolation specificity. The *Aspergillus* genus was isolated from both hosts only on MDA; a larger amount of isolates identified as *Diaporthe/Phomopis* were found using both hosts and both media.

**Table 1:** **Endophytic isolates using two different media (PDA and MDA) from guarana (G),** ***Paullinia cupana*** **(G-PDA and G-MDA) and olive (O)** ***Olea europaea*** **(O-PDA and O-MDA)**

Genera	Isolates			
	G-PDA	G-MDA	O-PDA	O-MDA
*Aspergillus*	0	1	0	1
*Bionectria*	0	1	0	0
*Botrysphaeria*	1	2	0	0
*Cladosporium*	0	2	0	0
*Cochliobolus*	0	2	0	0
*Colletotrichum*	4	3	1	2
*Coniosporium*	1	2	0	0
*Daldinnia*	0	0	1	0
*Diaporthe Phomopsis*	12	8	20	22
*Guignardia*	0	6	1	0
*Nigrospora*	0	0	1	0
*Pestalotiopsis*	0	3	0	0
*Phanerochaete*	0	1	0	0
*Pleosporales*	0	1	0	0
*Sordariomycetes*	2	0	0	0
*Xylaria*	8	1	2	2
**Diversity parameters** ^**a**^				
**S**	6	13	6	4
**N**	28	33	26	27
**D**	1.50	3.40	1.53	0.91
**H'(loge)**	1.43	2.29	0.90	0.67
**1-Lambda'**	0.73	0.89	0.41	0.34

^a^Diversity parameters: S–total taxon, N–total individuals, D–species richness (Margalef), H"(loge)–Shannon and 1-lambda'–Simpson diversity indexes of endophytic fungal communities that were isolated from guarana (G) and olive plants (O) using both BDA and MDA media.

In guarana, only six genera were identified using PDA and 13 genera were identified using MDA; from the olive fungal community, six genera were isolated using PDA and 4 genera using MDA. These data show that the greatest diversity was found when the guarana tree and MDA were used. Both the Shannon (H"log_e_) and Simpson (1-lambda') diversity indices were highest in G-MDA (Table 1).

We used the PAST software to compare the diversity indices (*t* test) for PDA and MDA to obtain significant values (*p* < 0.05) for PDA and MDA separation when isolating fungi from guarana. The significant values for the media types did not differ when isolating fungi from the olive plant. Guarana and olive have distinct communities that are independent of the media source (Table 2).

**Table 2:** **Diversity** ***t*** **test on endophytic fungi that were isolated from guarana and olive plants using PDA and MDA media**

PDA ***vs*** MDA media			
	Variance	***p***	t
**Whole sample**	0.49	0.003**	-3.03
**Guarana**	0.09	0.0017**	-3.27
**Olive**	0.02	0.29 (ns)	-1.06
**Guarana** ***vs*** **Olive host**			
	**Variance**	***p***	**t**
**Whole sample**	0.21	7.3E-06**	5.23
**PDA**	0.07	0.035*	2.169
**MDA**	0.198	0.00011**	4.14

ns–non-significant.

*Differ statistically with *P* >0.05.

**Differ statistically with *P* >0.01.

**Figure 1:**
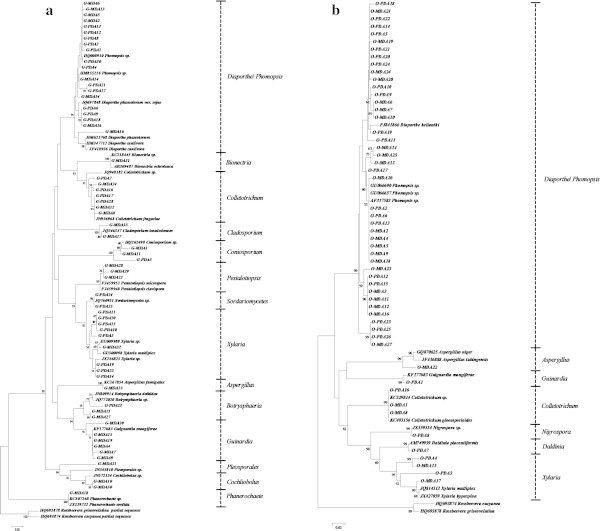
**Phylogram of isolated fungal communities from guarana and olive trees.** The construction of Neighbor-joining tree was based on the Jukes and Cantor model obtained from an analysis of the rDNA-ITS sequences of 62 fungal endophytes that were isolated from guarana **(a)** and 53 that were isolated from olive plants **(b)**. Reference sequences from GenBank were used to compare the relationships between isolates. The basidiomycete *Rossbeevera* spp. was used as the out-group. The sequences obtained in this work are in bold. The bootstrap values were *n* = 1,000 replicates.

Guarana has a more diverse fungal community that is better assessed when MDA is used as media source. We observed the separation of some isolates within each genus, and this separation was dependent on the host. A media-influenced separation of isolates was not observed. However, some other notable observations were made. The *Xylaria* fungi were obtained from both plant and media, but from guarana G-MDA32 did not cluster with the other *Xylaria* when using PDA (Figure 1). A similar result was found among the *Botrysphaeria* fungi. The isolate G-PDA23 did not cluster with G-MDA25 or G-MDA27, suggesting that the media type influenced the selection of isolates within a genus. The isolates from olive did not differ with the media source (Figure 1b).

### PCA and similarity analysis

**Figure 2:**
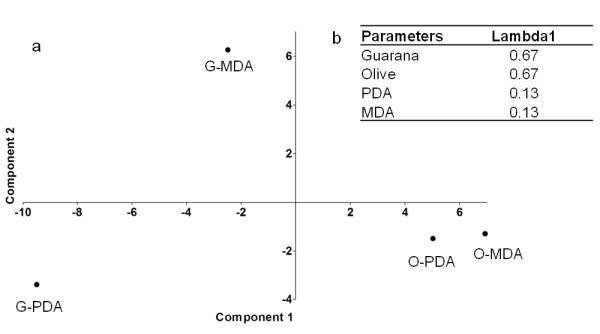
**Multivariate analysis of the isolated fungal from guarana (G) and olive plants (O) on MDA and PDA media. (a)** Principal components analysis (PCA) **(b)** Statistical parameters calculated using multivariate analyses with inclusion of a Monte Carlo permutation test. The values for Lambda 1 indicate the amount of variance that is explained by each parameter.

**Figure 3:**
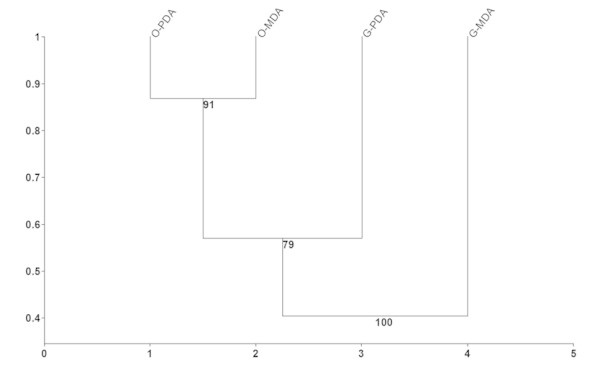
**Clustering of the similarity of endophytic fungal communities from guarana (G) and olive plants (O) isolated using PDA and MDA.** The bootstraps are the means of 1,000 repetitions.

DCA analysis of the species distribution revealed gradient lengths of 1.592 in size, which showed a linear distribution of data, justifying the use of redundancy analysis of these isolated fungal communities. The host was the most important factor affecting the composition of the fungal communities in both media, as can be observed in Figure 2a (its vector is most closely correlated with the first axis of the ordination diagram). This effect was significant, and the Lambda 1 values were 0.67 and 0.13 for the host and media, respectively (Figure 2b). Using clustering analysis, we observed a clear grouping of the isolates from olive (Figure 3), which shows a higher similarity between the fungal communities in a host than those in a media.

## Discussion

The present work reported the isolation, identification and characterization of cultured endophytic fungi from leaves of two climatically distinct plants (guarana and olive) using two types of starch source media (PDA and MDA).

Independently of the host plant, the MDA medium showed to be an alternative medium. The influence of the media on fungal growth is commonly described in laboratory procedures. Silva and Teixeira (2012) characterized *Fusarium solani* isolates from cassava roots under different media and light conditions and observed a better mycelial fungal growth on PSA (Potato Sucrose Agar). The MDA medium has also shown better mycelia growth and poorer spore formation compared with those in the PDA medium (Sia 2012). This could result in the increased isolation of some fungal species in MDA compared with PDA.

Over recent decades, potatoes have been a more common starch source for use in fungal growth-promoting media due to their complex carbohydrates, which improve the reproduction of mitosporic fungi (Lukens 1963; Strandberg 1987). However, new alternatives have been developed. Fungal isolation using the MDA medium demonstrated its viability as an alternative due to its ability to support a greater diversity and number of isolates of fungal communities (especially from a tropical host).

The MDA medium presents economic advantages over potatoes in tropical countries because cassava is cheaply and easily found in these countries. Moreover, most potato plantations are submitted to agrochemical treatment, which often results in fungal growth inhibition, whereas a traditional cassava plantation is generally free of agrochemicals.

*Coniosporium*, *Phaerochaete* and *Biocnectria* were only isolated using MDA and guarana. The isolation of these genera is not common in tropical plants. *Phanerochaete* was recently isolated from the plant *Andrographis paniculata* (Biji et al. 2011), and *Bionectria* was recently isolated from leaf tissues of *Sonneratia caseolaris* (Ebrahim et al. 2012). All of these genera (and others found in guarana) have interesting characteristics that are otherwise never related with tropical plants. For example, some species of the genus *Coniosporium* occur primarily on dead or living plant material or as a biodegradative fungus on aging monuments (Sert and Sterflinger 2010). The white rot fungi, genus *Phanerochaete,* have been reported to be highly lignolytic and cellulolytic. The genus *Bionectria* is important in decomposing plant debris and has other useful properties (Rossman 1996). Thus, these fungi may play important roles in decomposing plant material that later provides nutrition to their plant hosts in the Amazon.

*Colletotrichum* and *Diaporthe/Phomopsis* were isolated in both plants and both media. *Colletotrichum* are often symptomatic pathogens that have been isolated from numerous plant species. However, they can also be asymptomatic endophytes. *Colletotrichum* spp. can cause anthracnose in olive and guarana trees, which results in severe production loss (Duarte et al. 1995; Talhinhas et al. 2005). This genus is also a common tropical fruit plant pathogen in avocado, banana, citrus, mango, papaya, passion fruit, guava and cashew cultivation (Afanador-Kafuri et al. 2003; Figueirêdo et al. 2012). However, the leaves sampled in present study were healthy and without anthracnose symptoms. *Colletotrichum* is known for its production of the anticancer drug taxol (Gangadevi and Muthumary 2008). The medicinally and industrially important extra cellular lipase (Balaji and Ebenezer 2008), an antimicrobial metabolite (colletotric acid (Zou et al. 2000)) and colletotric acid (an 11*β*-hydroxysteroid dehydrogenase type 1 (11*β*-HSD1) inhibitor (Aoyagi et al. 2008)) were also isolated from these fungi genera. The cosmopolitan lifestyle of this interesting fungal group is likely a consequence of the high morphology and genetic variation that happens in the species of this genus (Sutton 1992).

Interestingly, a large amount of endophytic isolates found primarily in olive plants were identified as *Diaporthe/Phomopsis*, suggesting that this genus is a dominant member of the guarana and olive plant endophytic community. Silva et al. (2005) studied the bioactive metabolites that are produced by endophytic fungi in the cassia tree (*Cassia spectabilis*, family Leguminosae) and observed that the genus *Phomopsis* potentially produces many biologically antifungal metabolites and especially those against the phytopathogenic fungi *Cladosporium sphaerospermum* and *C. cladosporioides*. Nithya and Muthumary (2010) verified that the genus *Phomopsis* has a strong antibacterial activity and is a rich source of secondary metabolites. *Phomopsis* spp. were also isolated from Amazonian toxic plants (Souza et al. 2004), and further investigation is needed regarding its role as an endophyte of tropical and temperate host trees.

PDA and MDA had the same performance regarding olive fungi isolation but PDA presented less fungi genera diversity compared to MDA when used with guarana. To study the composition of the endophytic community of olive plants (*O. europaea* L.) from two sites in Sicily (Italy), Ferraro et al. (2008) observed that the number of fungal isolates differed between the sampled sites, using the malt extract agar medium (MEA), the prevailing fungal genera were *Alternaria*, *Cladosporium*, *Diplodia*, *Phoma*, *Septoria* and *Stemphylium*. When comparing the isolates from the two sites, the authors observed that the endophytic assemblages in olive trees had a similar composition at both of the sampled sites. However, none of the genera described by the authors were identified in the present study. The endophytic fungal species detected in plants may be influenced by many factors including the type of tissue sampled, when the plant was assayed, and perhaps the climate and location in which they grew (Impullitti and Malvick 2013), as well as the media used to isolate species. However, the influence of the media may be dependent on the accessed host. Using similarity data, we observed strong clustering between the PDA and MDA fungal communities that were isolated from olive plants. The similarity between separate guarana fungal communities that were isolated on either PDA or MDA was not significant. This suggests that media exerts more influence on fungal selection for diverse fungal communities such as those in tropical plants.

Although the early endophytic fungi studies were performed on temperate climate plant species, some studies (Arnold et al. 2000; Frohlich and Hyde 1999; Lodge et al. 1996) demonstrated that tropical plant species are host to a greater fungal biodiversity. Our data proved that guarana and olive plants presented distinct fungal communities. However, few studies have quantified the endophyte colonization patterns in tropical plants (Arnold et al. 2000). Most of the results thus far indicate that tropical endophytes themselves may be hyper-diverse; therefore, tropical host endophytes constitute a species-rich ecological assemblage of fungi. The results from other studies corroborate our hypothesis and demonstrate that using alternative media should improve the investigation and elucidation of these organisms.

The clustering of PDA and MDA isolated endophytic fungal communities from guarana and olive plants showed a higher fungal community similarity according to host than according to medium. The present data demonstrated that most of the variance in fungal species was explained by the host. However, the media likely influence the selection some isolated species. According to Arnold et al. (2000), tropical forests contribute substantially to fungal diversity, and considering statements by Carnaúba et al. (2007), the media composition is a determining factor in fungal growth.

## Conclusions

The results of the present study demonstrated the feasible use of MDA as an alternative medium source to fungal growth. The important role of the media in fungal isolation from two common trees in tropical and subtropical climates was also proved. The MDA media showed more diverse results that demonstrate its potential as an alternative starch source for laboratorial fungal growth. The implementation of this media for the isolation of endophytic fungi should improve the number of isolates and increase access to a greater diversity of the fungal community.

## Methods

### Culture media

Two culture media were used: Potato Dextrose Agar (PDA) was prepared in our laboratory, and consisted of potato extract (200 g), dextrose (20 g), agar (15 g), and distilled water to a final volume of one liter with a pH of 6.5. Manihot Dextrose Agar (MDA) was prepared using the same method as PDA but with 200 g of cassava instead of potato. To prepare the potato and cassava extracts, 200 g of each plant tuber was cut into pieces and heated in distilled water for 30 min. The infusion was filtered, dextrose was added and the pH was adjusted to 6.5. After agar addition, the media were autoclaved for 15 min at 121°C (Sia 2012).

### Fungal isolation

Endophytic fungi were isolated from two independent experiments, two plant species: olive (*O. europaea*), which is a common plant in temperate and subtropical areas, and guarana (*P. cupana*), which is a tropical plant native to the Amazon region. A random sample from each tree consisting of approximately ten healthy leaves was taken for *P. cupana* from the Brazilian Amazon town of Maués (03º 23′ 01″ S, 57º 43′ 07″ W) and for *O. europaea* from the campus of the Agriculture School, University of São Paulo, Piracicaba, Brazil (22° 43′ 30″ S, 47° 38′ 51″ W). After removal, the leaves were placed in plastic bags and transported to the laboratory. They were used within 24 h of their removal. All of the leaves were washed with running tap water. The plant tissues were rinsed with 70% ethanol for 1 min and their surfaces were disinfected with a sodium hypochlorite solution (3% available Cl^–^) for 3 min. They were again rinsed once for 30 s in 70% ethanol and then rinsed twice in sterile distilled water according to Araújo et al. (2001). The efficiency of the disinfection process was checked by plating aliquots of the sterile distilled water that was used in the final rinse on Potato Dextrose Agar (PDA) and Manihiot Dextrose Agar (MDA). After surface disinfection, each leaf was cut into square fragments (4–6 mm) that were placed onto PDA or MDA that contained tetracycline (50 μg/mL) and streptomycin (50 μg/mL) to suppress bacteria growth. From each experiment, for each plant species, 17 plates from each media were used. A total of 102 leaf fragments were used for each plant host (six fragments per Petri dish). After 30 days of incubation at 28°C, the hyphal tips of each morphologically different mycelium that emerged from a leaf fragment were subcultured and transferred to PDA or MDA. These tips were purified and then transferred to slants for later identification. The preservation of the isolates was performed according to the Castellani method (Castellani 1967). The endophyte incidence (EI) was calculated as: the percentage of pieces that showed fungal growth/total number of pieces that were screened × 100.

### Molecular identification

The DNA extraction was performed on 150 purified fungal strains according to the method used by Raeder and Broda (1985). The amplification of ITS1-5.8S-ITS2 rDNA was performed using the primers ITS1 (5′TCCGTAGGTGAACCTGCGG′3) and ITS4R (5′TCCTCCGCTTATTGATATGC′3) in a Peltier Thermal Cycler PTC200 (MJ Research Inc, Watertown, MA). The PCR mix contained 3 ng of template DNA, 0.4 μM of each primer, 3.7 mM of MgCl_2_, 250 μM of each dNTP, 2 U of *Taq* DNA polymerase (Invitrogen, Brazil), 50 mM of Tris–HCl (pH 8.4), and 20 mM of KCl to achieve a 50 μL final volume. The PCR consisted of 35 cycles (denaturing for 30 s at 94°C, primer annealing for 30 s at 55°C and extension for 30 s at 72°C). Negative controls containing all reagents except for the genomic DNA were prepared in each PCR reaction. The PCR product was analyzed using electrophoresis on 1.0% w/v agarose gels, stained with ethidium bromide and visualized under UV light.

The amplified fragments were purified using polyetilene glicol (Sambrook and Russell 2001) and sequenced by the Human Genome Research Center, Institute of Biosciences, University of São Paulo, São Paulo, Brazil with the ABI 3730 DNA Analyzer (Applied Biosystems) according to the manufacturer’s instructions.

### Sequence assembly and alignment

Consensus sequences were manually aligned using the MEGA v5.1 software (Tamura et al. 2011) and BioEdit 7.0 (Hall 1999) to insert gaps. rDNA-ITS sequences were aligned on the basis of similarity by means of the sequence alignment software CLUSTAL-W 1.7 (Thompson et al. 1994). The identification of sequences was realized using blastn and the nucleotide collection (nr/nt) database (http://www.ncbi.nlm.nih.gov/blast/).

The neighbor-joining analyses were conducted using Megav5.1 with the branch support values obtained after 1,000 heuristic searches for pseudo replicates. The sequences presented in this study (a total of 114 sequences) were submitted to GenBank (accession numbers JX944069-JX944183). The many diversity parameter indices were: S–total profile, N- profile value, d–species richness (Margalef), and H" (loge) - Shannon and 1-lambda'–Simpson diversities were obtained using 'Primer 6’ (Plymouth Marine, Primer, United Kingdom).

### Data analysis

The correlations between individual isolates and environmental variables (plant species and media used) were determined using multivariate analysis with Canoco for Windows 4.5 software (Biometris, Wageningen, The Netherlands) and following the same procedures as previously described (Andreote et al. 2009; Ramete 2007; Ter Braak and Šmilauer 2002). Briefly, a detrended correspondence analysis (DCA) was performed to calculate the gradient distribution of the genera in all of the evaluated treatments. In the case of normal distribution in fungal community identification (gradient in the first axis < 4.0), the data were analyzed using Redundancy Analysis (RDA). For statistical analyses of correlations between genera and variable factors (host plant and media), a Monte Carlo permutation test was performed and was based on 499 unrestricted permutations. In addition to the P-values, the values of Lambda 1 were obtained to quantify the amount of variance that could be explained by each variable factor. The data were also analyzed using principal component analysis (PCA). The axis values showed the percentage variance of each accessed fungal community.

We also performed similarity clustering using the PAST program (Hammer et al. 2001) to prove that there were significant differences between the communities (Clarke and Ainsworth 1993). The Bray-Curtis model was adapted to the present data.
